# The Role of Depressive Symptoms and Physical Activity Levels in Mediating the Association Between HIV Status and Neurocognitive Functions Among Individuals Aged at Least 50 Years in China: Cross-sectional Study

**DOI:** 10.2196/32968

**Published:** 2022-08-19

**Authors:** Pei Qin, Jianmei He, Xue Yang, Siyu Chen, Xi Chen, Hui Jiang, Ada Wai Tung Fung, Zixin Wang, Joseph Tak Fai Lau

**Affiliations:** 1 Shenzhen Qianhai Shekou Free Zone Hospital Shenzhen China; 2 Jockey Club School of Public Health and Primary Care, Faculty of Medicine The Chinese University of Hong Kong Hong Kong Hong Kong; 3 Hunan Provincial Center for Disease Control and Prevention Changsha China; 4 Department of Applied Social Sciences The Hong Kong Polytechnic University Hong Kong Hong Kong; 5 Affilliated Kangning Hospital of Wenzhou Medical University Wenzhou Medical University Wenzhou China; 6 School of Mental Health Wenzhou Medical University Wen Zhou China; 7 School of Public Health Zhejiang University Zhejiang China

**Keywords:** neurocognitive performance, HIV sero-status, depressive symptoms, level of physical activity, mediation effects, HIV, depression, physical activity, neurocognitive, mental health, public health

## Abstract

**Background:**

Neurocognitive impairments are prevalent among older people in China. It is more problematic among older people living with HIV.

**Objective:**

This study aims to compare neurocognitive performance between older people living with HIV and HIV-negative controls, and to explore whether the association between HIV status and neurocognitive performance was mediated by depressive symptoms and level of physical activity.

**Methods:**

A cross-sectional study was conducted in Yongzhou, China. All people living with HIV aged ≥50 years listed in the registry were invited. Frequency matching was used to sample HIV-negative controls from the general population according to the distribution of age, sex, and years of formal education of older people living with HIV. A total of 315 older people living with HIV and 350 HIV-negative controls completed the face-to-face interview and comprehensive neuropsychological assessment of seven domains (learning, memory, working memory, verbal fluency, processing speed, executive function, and motor skills).

**Results:**

As compared to HIV-negative controls, older people living with HIV performed worse in global score and all seven domains (*P*<.05). HIV infection was associated with higher depressive symptoms (*P*<.001) and lower level of physical activity (*P*<.001). Depressive symptoms and physical activity were negatively correlated (*P*<.001). Depressive symptoms and level of physical activity mediated the association between HIV status and global *z*-score and four domain *z*-scores of neurocognitive performance (learning, memory, verbal fluency, and processing speed).

**Conclusions:**

Change in mental health and physical activity after HIV infection may partially explain why older people living with HIV are more susceptible to neurocognitive impairment. Promoting mental health and physical activity are potential entry points to slow down the progress of neurocognitive impairment among older people living with HIV.

## Introduction

The Centres for Disease Control and Prevention specify the age of older people living with HIV as 50 and above [1,2]. Globally, the size of older people living with HIV has been increasing rapidly due to the advancement in the efficacy and coverage of antiretroviral therapy (ART) [3-5]. Take the United States as an example; the proportion of people living with HIV aged 50 years or above was about 45% in 2014 and was projected to exceed 75% in 2030 [6]. In China, such proportion has increased by 20% from 2001 (1.94%) to 2011 (21.1%) [7]. Older people living with HIV are more likely to have aging-related conditions due to HIV infection [8,9].

HIV infection is a risk factor of neurocognitive impairment. Studies showed older people living with HIV had poorer neurocognitive function as compared to HIV-negative individuals [10-12]. Neurocognitive impairment is prevalent and consequential among older people living with HIV [13]. The Central Nervous System HIV Antiretroviral Therapy Effects Research study reported that nearly half of people living with HIV suffered from neurocognitive impairment [13]. Other studies showed that 37.0%-69.9% of older people living with HIV in some western countries had such condition [14,15]. Neurocognitive impairment results in poorer adherence to ART, faster disease progression, poorer quality of life, and higher all-cause mortality among people living with HIV [16-20].

Mental health problems (eg, depression) are the most commonly reported comorbid conditions of people living with HIV [21]. Studies consistently showed that mental health problems are more common among older people living with HIV as compared to their younger counterparts [22]. Across countries, the prevalence of depression among older people living with HIV ranged from 39.1% to 60.5% [23-26]. There are potential reasons that may contribute to the elevated risk of mental health problems among older people living with HIV. Studies showed that age-related reduction in immune responses, impaired physical function, greater difficulties to cope with HIV-related stress, and reduced social support might contribute to or exacerbate existing mental health problems among older people living with HIV [22,25]. Studies suggest that depression is associated with brain vascular disease, which damages critical cortico-striatal circuits and results in neurocognitive impairment [27]. Depression is a strong risk factor of neurocognitive impairment among both general populations [28] and people living with HIV [11,29,30].

Physical activities are beneficial and recommended for older people living with HIV [31]. Studies showed that higher level of physical activity was associated with lower odds of neurocognitive impairment among older people living with HIV [32,33]. However, older people living with HIV encountered more barriers to perform physical activities as compared to their HIV-negative counterparts. Many barriers are related to their HIV-positive status. First, older people living with HIV are more likely to develop age-related chronic conditions, including cardiovascular diseases, lung diseases, and cancer, which have been shown to negatively affect physical function and the ability to perform physical activities [34]. Second, side effects of ART, reduced social support due to HIV infection, and social stigma or discrimination also hinder older people living with HIV to do physical activities [35,36]. Therefore, there is a large body of literatures showing that older people living with HIV, even when virally suppressed by ART, exhibit much lower level of physical activity when compared to age-matched HIV-negative controls [37-39]. A recent study showed that 86% of older people living with HIV did not achieve the recommended physical activity level, as measured by accelerometer [40].

Given depressive symptoms and physical activities were associated with both HIV infection and neurocognitive function among older people living with HIV, it is possible that depressive symptoms and level of physical activity would mediate the association between HIV infection and neurocognitive function. Identifying mediators is important to explain the difference in neurocognitive function between older people living with HIV and their HIV-negative counterparts. The path analysis has significant implications for interventions, and health workers can alleviate the adverse effect of HIV infection on neurocognitive function among older people. To our knowledge, no study has tested such a mediation hypothesis.

In this study, we compared neurocognitive performance (global score and seven domains), depressive symptoms, and level of physical activity between older people living with HIV and HIV-negative controls matched by age, gender, and education in China. We further test the hypothesis that depressive symptoms and level of physical activity would mediate the association between HIV infection and neurocognitive performance.

## Methods

### Study Design

A cross-sectional study was conducted in Yongzhou city in southern China from March to December 2017. The city has a population size of 6.3 million and a disposable income per capita of 15,292 RMB (US $2438) in 2015 (median in China was 22,408 RMB [US $3573]). The city consists of 2 districts and 9 counties. One district (Lingling) and 4 counties (Ningyuan, Lanshan, Qiyang, and Dao) were conveniently selected as the study sites.

### Participants

Participants were older people aged ≥50 who received confirmatory HIV diagnosis. Exclusion criteria included the following: (1) severe hearing loss or impaired vision observed by the interviewers, (2) history of brain injury with or without loss of consciousness (>30 minutes), brain tumor, stroke, or brain opportunistic infection; and (3) major psychiatric illnesses (schizophrenia and bipolar disorder). The second and third exclusion criteria were self-reported information or based on clinicians’ assessments according to their medical records.

### Data Collection

Provincial or local Centres for Disease Control and Prevention and HIV clinics of local hospitals facilitated the recruitment of older people living with HIV. These institutions serve all people living with HIV in the selected district and counties and are responsible for HIV testing and diagnosis, CD4 (cluster of differentiation 4) testing, and management of the ART. The staff of these institutions contacted all older people living with HIV listed in the registries of the selected district and counties. With verbal consent, they screened prospective participants’ eligibilities to join the study, briefed them about the purpose and logistics of the study, and invited them to be interviewed at the HIV clinics. The participants were assured that their information would be kept confidential, and refusal to participate would not affect their right to use future services. Written informed consent was obtained before conducting the face-to-face interviews and the neurocognitive assessments. The whole process took 1.5-2 hours to complete, with breaks in between. Upon completion, a monetary incentive of 50 RMB (US $7.96) was given to the participants for their time.

HIV-negative controls were recruited from general population in the corresponding study sites. In these study sites, health service centers provide comprehensive health-related services to local residents. These centers keep contact information of all residents living in the area. In this study, these health service centers facilitated the recruitment of HIV-negative controls. We used frequency matching to sample HIV-negative controls according to the distribution of age (SD 3 years), sex, and years of formal education of older people living with HIV. Staff of the health service centers approached households in person or via telephone, screened eligibility, and invited eligible residents to participate. The procedures for obtaining written informed consent and conducting face-to-face interview and neurocognitive assessment were the same as those for older people living with HIV. These participants were then invited to take a finger-prick HIV rapid test (Alere Determine HIV-1/2 rapid HIV screening test, Alere Inc, Watham, MA, United States; sensitivity: 99.75%, specificity: 100%).

### Ethics Approval

Ethics approval was obtained from the Survey and Behavioral Research Ethics Committees of the Chinese University of Hong Kong and the joint Chinese University of Hong Kong—New Territories East Cluster Clinical Research Ethics Committee (Ref# 2017.550).

### Neurocognitive Assessments

The comprehensive neuropsychological test battery was used in this study. It comprised of neuropsychological tests of seven domains. Learning and memory were assessed by the Chinese Auditory Verbal Learning Test [41]. Attention or working memory was measured by the digit span (forward and backward) and visual span (forward and backward) methods [42]. Information processing speed was assessed by the performance on the Chinese Trail Making Test Part A [43]. Executive function was assessed by the Chinese Trail Making Test Part B [44]. Verbal fluency was assessed by the category verbal fluency tests (animal, fruit, and vegetable) [45]. Motor skills were evaluated by the grooved pegboard for both dominant hand and nondominant hand [46]. These tests were commonly used in studies targeting people living with HIV [47] and were validated in the Chinese population [48].

PQ received intensive training on neurocognitive assessment from an experienced and practicing neuropsychologist. She completed neurocognitive assessments for ten older people living with HIV in the study sites. All practice assessments were audiotaped and sent to the neuropsychologist for review and competence assessment, which were found to be satisfactory. The first author then conducted a 2-week training workshop including guided practice and competence assessment for 4 other interviewers. They were deployed in fieldwork after they achieved satisfactory level of competence. During the first 2 weeks of fieldwork, PQ supervised neurocognitive assessments conducted by these 4 interviewers and provided individual feedback.

Raw scores of the aforementioned seven domains were transformed into standardized *z*-score, based on the mean and SD of the HIV-negative controls using the following formula: z-score = (raw test score – mean test score among HIV-negative controls) / SD of the test score among HIV-negative controls. Domain *z*-score was calculated by averaging the *z*-scores of tests in each domain, while global *z*-score was calculated by averaging the seven-domain *z*-scores. The same approach to calculate the domain and global score for neurocognitive performance has been used in published studies [49-51].

### Measurements

#### Depressive Symptoms

Depressive symptoms were assessed by the 20-item validated Chinese version of the Center for Epidemiological Studies-Depression scale (CES-D-20) [52,53]. This scale has been used among people living with HIV in China [54]. Scores of CES-D-20 range from 0 to 60, with higher scores indicating more severe depressive symptoms. In this study, Cronbach alpha of the CES-D-20 was .93.

#### Physical Activities

The 7-item International Physical Activity Questionnaire was used to measure walking as well as moderate- and vigorous-intensity activities in the past week [55]. Physical activity metabolic-equivalent tasks (METs) per week were computed [55]. High physical activity level was defined as (1) vigorous-intensity activity on at least 3 days and accumulating at least 1500 MET minutes per week, or (2) at least 5 days of any combination of walking and moderate-intensity or vigorous-intensity activities, achieving a minimum total physical activity of at least 3000 MET minutes per week. Moderate level was defined as meeting any one of the following criteria: (1) at least 3 days of vigorous activity of at least 20 minutes per day, (2) at least 5 days of moderate-intensity activity or walking of at least 30 minutes per day, or (3) at least 5 days of any combination of walking and moderate-intensity or vigorous-intensity activities, achieving a minimum total physical activity of at least 600 MET minutes per week. Individuals who did not meet the criteria for moderate or high levels of physical activity were considered as those with low physical activity or inactive.

#### Potential Confounders

Sociodemographic characteristics of age, sex, years of formal education, marital status, personal annual income, and living arrangement (whether living alone or not) were obtained.

Blood pressure was measured twice at 5-minute intervals in the right arm and in the sitting position by some nurses or doctors with a mercury sphygmomanometer. Systolic and diastolic blood pressure were calculated by averaging the 2 measurements. The use of antihypertensive drugs was asked in the questionnaire. Hypertension was defined as systolic blood pressure of ≥140 mmHg or diastolic blood pressure of ≥90 mmHg or self-reported antihypertensive drugs use. Self-reported diabetes was determined by a positive response to the question “Have you ever been told by a health professional that you have diabetes?”. Similar questions were used to measure the presence of hyperlipidemia, myocardial infarction, coronary heart disease, cerebrovascular disease, hepatitis B virus infection, hepatitis C virus infection, chronic bronchitis, chronic obstructive pulmonary disease, chronic liver disease, chronic kidney disease, peptic ulcer, stroke, cancer, peripheral vascular diseases, and connective tissue disease.

The participants were asked whether they are taking other medications, including diabetes medication, lipid-lowering drugs, aspirin, warfarin, drugs for heart disease, antidepressants, antidementia drugs, and nonsteroidal anti-inflammatory drugs. Two composite variables were constructed in this study by counting the number of affirmative item responses reflecting the number of chronic conditions and number of medications.

### Statistical Analysis

Descriptive statistics were presented. Between-group comparisons (depressive symptoms, physical activity level, and potential) were performed using the chi-square test or independent samples 2-tailed *t* test as appropriate. Potential confounders were controlled when comparing the differences in raw scores of neurocogitive tests and global- or domain-specific *z*-scores between older people living with HIV and HIV-negative controls using multivariable linear regression. Crude *P* values and adjusted *P* values were presented.

**Figure 1:**
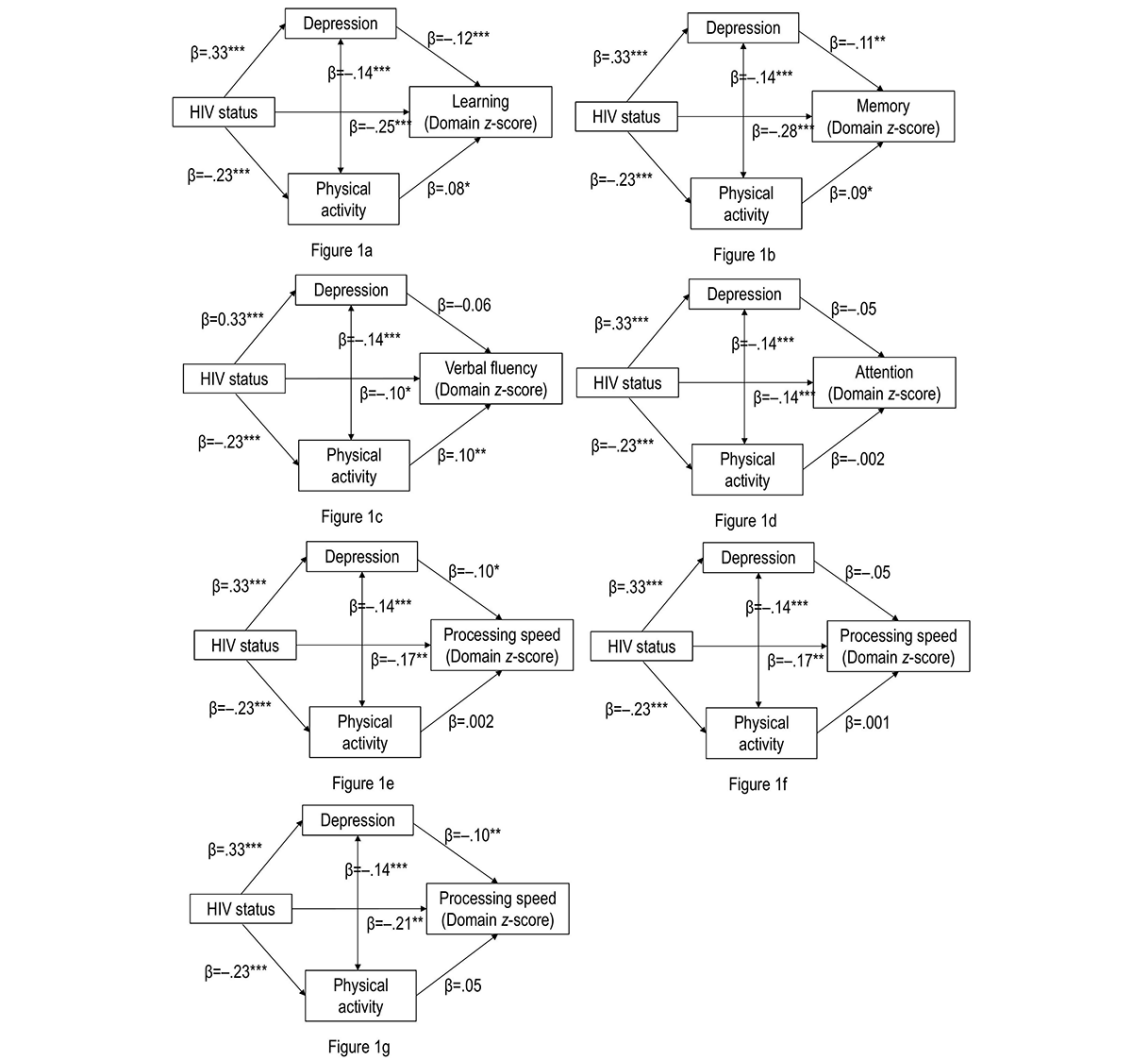
Mediation effects of physical activity and depressive symptoms in the association between HIV status and cognitive function (*z*-score); *: *P*<.05; **: *P*<.01; ***: *P*<.001. The path analysis presented the standardized regression weights and *P* value of each path.

Path analysis was conducted to test the mediation model (Figure 1). HIV status was used as independent variable, while raw score of a neurocognitive test, or global or a domain *z*-score was included as a dependent variable in each mediation model. Standardized path coefficients (*β*) and unstandardized path coefficients (B) were reported. Bootstrapping analyses tested the mediation hypotheses. The 95% CIs of the indirect effects would be obtained from 5000 bootstrap samples. A statistically significant mediation effect would be observed when the CI did not include zero. SPSS 21.0 for Windows and AMOS 17.0 (IBM Corp) were used for data analysis; the level of significance was set to *P*<.05.

## Results

### Descriptive Statistics

Of the 433 eligible older people living with HIV invited, 83 (19.2%) refused to join the study due to lack of time or interest. The response rate was 80.8% (350/433). Among the 350 participants consented to join the study, 14 (4%) and 21 (6%) did not complete the face-to-face interview and neurocognitive assessments, respectively; 315 (90%) completed both parts. Out of 434 controls being invited, 350 (80.6%) completed the face-to-face interview and neurocognitive assessments. None of the controls were screened to be HIV positive.

The mean age of the older people living with HIV was 61.3 (SD 6.8) years; 73.0% (230/315) were male; 52.1% (164/315) had an education level of primary school or below. Most of them were of Han ethnicity (307/315, 97.5%), were married (215/315, 68.3%), had an annual personal income of no more than 10,000 RMB (US $1507; 163/315, 52.1%), and were living with someone else (224/315, 71.1%). The number of chronic conditions and mediation use was 1.3 (SD 1.2) and 0.3 (SD 0.6), respectively. 79.4% (250/315) had received HIV diagnosis within 4 years, 56.4% (177/315) had a current CD4 level of <350 cells/µL, and 59.0% (186/315) had a CD4 nadir lower than 350 cells/µL. Of the 308 participants who were on ART, 21.9% (67/308) reported any missing dose in the last month, and 60.7% (187/308) were taking efavirenz.

Distributions of age (*P*=.62), sex (*P*=.53), and years of formal education (*P*=.48) did not differ between older people living with HIV and HIV-negative controls, reflecting successful matching. However, HIV-negative controls were less likely to be widowed (*P*<.001) and living alone. The between-group difference in mean number of chronic conditions was of marginal statistical significance (*P*=.07; Table 1).

**Table 1:** Characteristics of older people living with HIV and HIV-negative controls.

Characteristics				Older people living with HIV (n=315), mean (SD)		HIV-negative controls (n=350)		*P* values
**Sociodemographics**								
	Age (years), mean (SD)			61.3 (6.8)		61.1 (6.4)		.62
	**Sex, n (%)**							.53
		Male	230 (73.0)		257 (73.4)			
		Female	85 (27.0)		83 (23.6)			
	Years of education, mean (SD)			5.7 (3.6)		5.5 (3.4)		.48
	**Ethnicity, n (%)**							.09
		Han	307 (97.5)		347 (99.1)			
		Others	8 (2.5)		3 (0.9)			
	**Marital status, n (%)**							<.001
		Married	215 (68.3)		303 (86.8)			
		Widowed	72 (22.9)		29 (8.3)			
		Divorced or single	28 (8.9)		17 (4.9)			
	**Annual personal income (RMB), n (%)**							.59
		≤10,000^a^	163 (52.1)		187 (54.2)			
		>10,000	150 (47.9)		158 (45.8)			
	**Living alone, n (%)**							<.001
		No	224 (71.1)		310 (88.8)			
		Yes	91 (28.9)		39 (11.2)			
**Depressive symptoms**								
	Score of CES-D-20^b^, mean (SD)			17.4 (13.0)		9.4 (9.4)		<.001
**Level of physical activity, n (%)**								<.001
		Low	61 (19.4)		32 (9.2)			
		Moderate	140 (44.4)		115 (32.9)			
		High	114 (36.2)		202 (57.7)			
**Presence of chronic conditions, n (%)**								
	Hypertension			162 (51.4)		196 (56.0)		.24
	Diabetes			23 (7.3)		24 (6.9)		.81
	Hyperlipidemia			12 (3.8)		12 (3.4)		.84
	Chronic bronchitis			26 (8.3)		21 (6.0)		.29
	Chronic obstructive pulmonary disease			7 (2.2)		4 (1.1)		.27
	Cerebrovascular disease			22 (7.0)		24 (6.9)		.94
	Coronary heart disease			15 (4.8)		21 (6.0)		.48
	Myocardial infarction			1 (0.3)		3 (0.9)		.63
	Hepatitis B			23 (7.3)		2 (0.6)		<.001
	Hepatitis C			1 (0.3)		0 (0.0)		.47
	Liver dysfunction			15 (4.8)		3 (0.9)		.002
	Liver cirrhosis			5 (1.6)		0 (0.0)		.02
	Chronic kidney disease			12 (3.8)		6 (1.7)		.09
	Peptic ulcer disease			31 (9.9)		27 (7.7)		.33
	Cancer			15 (4.8)		6 (1.7)		.02
	Peripheral vascular disease			13 (4.1)		5 (1.4)		.03
	Connective tissue disease			26 (8.3)		23 (6.6)		.39
	Number of chronic conditions, mean (SD)			1.3 (1.2)		1.1 (1.2)		.07
**Use of medication**								
	Number of medication use^c^, mean (SD)			0.3 (0.6)		0.3 (0.7)		.12
**HIV-related disease characteristics**								
	**Duration since HIV diagnosis, n (%)**							
		<1 year	100 (31.7)		N/A^d^		N/A	
		1-3 years	150 (47.6)		N/A			
		≥4 years	65 (20.6)		N/A		N/A	
	**Most recent CD4^e^ count (cells/uL)**							
		<350	177 (56.4)		N/A		N/A	
		350-500	70 (22.3)		N/A		N/A	
		>500	67 (21.3)		N/A		N/A	
	**CD4 nadir, cells/μL**							
		<200	109 (34.6)		N/A		N/A	
		200-350	77 (24.4)		N/A		N/A	
		350-500	20 (6.4)		N/A		N/A	
		>500	8 (2.5)		N/A		N/A	
		Missing	101 (32.1)		N/A		N/A	
	**On ART^f^, n (%)**							
		Yes	298 (97.8)		N/A		N/A	
		No	7 (2.2)		N/A		N/A	
	**Missing of any ART doses in the last month (among those who were on ART; n=308), n (%)**							
		Yes	67 (21.9)		N/A		N/A	
		No	241 (78.1)		N/A		N/A	

^a^10,000 RMB= US $1507.

^b^CES-D-20: 20-item Center for Epidemiological Studies-Depression.

^c^The number of medications were constructed by counting the number of affirmative item responses on whether they are taking other medications, including diabetes medication, lipid-lowering drugs, aspirin, warfarin, drugs for heart disease, antidepressants, antidementia drugs, and nonsteroidal anti-inflammatory drugs.

^d^N/A: not applicable.

^e^CD4: cluster of differentiation 4.

^f^ART: antiretroviral therapy.

### Between-Group Differences in Neurocognitive Performance, Depressive Symptoms, and Physical Activities

After being controlled for age, sex, years of formal education, marital status, personal annual income, living arrangement (whether living alone or not), and number of chronic conditions and mediation use, older people living with HIV had poorer performance in all neurocognitive tests, with the exception of visual span—backward (*P*=.08). Older people living with HIV had poorer performance in all seven domains (*P*<.001 to *P*=.01) and global neurocognitive function (*P*<.001) compared with HIV-negative controls (Table 2).

As compared to HIV-negative controls, older people living with HIV had more severe depressive symptoms (CDS-D-20 score 17.4, SD 13.0 versus 9.4, SD 9.4; *P*<.001) and lower physical activity level (high physical activity level 36.2% versus 57.7%; *P*<.001; Table 1).

**Table 2:** Difference in raw scores of neurocognitive tests and z-scores of global or domain of neurocognitive performance between older people living with HIV and HIV-negative controls.

Neurocognitive domains		Older people living with HIV (n=315), mean (SD)	HIV-negative controls (n=350), mean (SD)	Crude *P* values^a^		Adjusted *P* values^b^	
**Learning**							
	CAVLT^c^–total learning (raw score)	30.41 (10.54)	36.65 (9.38)	<.001		<.001	
	Domain *z*-score^d^	–0.66 (1.12)	0 (1)	<.001		<.001	
**Memory**							
	CAVLT–delayed recall (raw score)	5.64 (3.06)	7.59 (2.85)	<.001		<.001	
	Domain *z*-score^d^	–0.68 (1.08)	0 (1)	<.001		<.001	
**Verbal fluency**							
	Animal (raw score)	12.97 (3.51)	13.45 (3.07)	.06		.02	
	Fruits (raw score)	8.98 (2.77)	9.65 (2.63)	.003		<.001	
	Vegetable (raw score)	10.60 (3.17)	11.34 (3.16)	.002		.001	
	Domain *z*-score^d^	–0.21 (0.85)	0 (0.77)	.001		<.001	
**Attention or working memory**							
	Digit span—forward (raw score)	7.87 (2.61)	8.64 (2.60)	<.001		<.001	
	Digit span—backward (raw score)	3.71 (1.83)	4.13 (2.75)	.02		.01	
	Visual span—forward (raw score)	6.69 (1.67)	7.07 (1.59)	.003		.004	
	Visual span—backward (raw score)	4.63 (2.03)	4.89 (1.78)	.08		.08	
	Domain *z*-score^d^	–0.21 (0.70)	0 (0.69)	<.001		.002	
**Processing speed**							
	CTMT-A^e^ (raw score)	22.82 (20.27)	16.40 (10.99)	<.001		<.001	
	Domain *z*-score^d^	–0.58 (1.84)	0 (1)	<.001		<.001	
**Executive function**							
	CTMT-B^f^ (raw score)	140.70 (123.69)	110.01 (94.46)	.001		.001	
	Domain *z*-score^d^	–0.32 (1.31)	0 (1)	.001		.001	
**Motor skills**							
	Dominant hand (raw score)	113.99 (48.68)	104.36 (43.55)	.02		.02	
	Non-dominant hand (raw score)	123.04 (101.07)	108.57 (38.65)	.01		.01	
	Domain *z*-score^d^	–0.30 (1.62)	0 (0.96)	.01		.01	
	Global cognitive *z*-score^g^	–0.36 (0.73)	0 (0.58)	<.001		<.001	

^a^*P* values obtained by univariate linear regression models.

^b^Adjusted for confounders—age, sex, years of formal education, marital status, personal annual income, living arrangement (whether living alone or not), number of chronic conditions, and mediation use.

^c^CAVLT: Chinese Auditory Verbal Learning Test.

^d^*z*-scores of individual tests were calculated by using the following formula: (raw test score – mean test score among HIV-negative control) / SD of test score among HIV-negative controls. Domain *z*-scores were calculated by averaging *z*-scores of the tests within the respective domain.

^e^CTMT-A: Chinese Trail Making Test Part A.

^f^CTMT-B: Chinese Trail Making Test Part B.

^g^Global *z*-scores were calculated by averaging *z*-scores in all tests used in this study.

### Testing the Mediation Hypotheses

The models fitted the data well (chi-square=136.33; degree of freedom=54; comparative fit index: 0.90 to 0.92; root mean square error of approximation=0.05; Table 3). After being controlled for potential confounders (age, sex, years of formal education, marital status, personal annual income, whether living alone or not, number of chronic conditions, and mediation use), path analyses showed that positive HIV status was associated with higher depressive symptoms (*β*=.33, *P*<.001) and lower level of physical activity (*β*=–.23, *P*<.001). Depressive symptoms and physical activity were negatively correlated (*β*=–.14, *P*<.001). Depressive symptoms were negatively associated with the global *z*-score (*β*=–.10, *P*=.002) and three domain *z*-scores, which were learning (*β*=–.12, *P*<.001), memory (*β*=–.11, *P*=.001), and processing speed (*β*=–.10, *P*=.02). The level of physical activity was positively associated with three domain *z*-scores, including learning (*β*=.08, *P*=.03), memory (*β*=.09, *P*=.01), and verbal fluency (*β*=.10, *P*=.01; Table 3 and Figure 1).

Significant indirect effects of HIV status were found on global *z*-score (*β*=–.06, 95% CI –0.10 to –0.03, *P*<.001) and four domain *z*-scores of learning (*β*=–.13, 95% CI –0.20 to –0.07, *P*=.001), memory (*β*=–.12, 95% CI –0.18 to –0.06, *P*=.001), verbal fluency (*β*=–.07, 95% CI –0.13 to –0.02, *P*=.002), and processing speed (*β*=–.10, 95% CI –0.22 to –0.02, *P*=.05; Table 3 and Figure 1).

Path analysis using raw scores of neurocognitive test as dependent variable and HIV status as independent variables were presented in Multimedia Appendix 1.

**Table 3:** Model fit and indirect effects of the proposed mediation model

Dependent variable	CFI^a^	Total effect, *β* (95% CI)^b^	Indirect effect, *β* (95% CI)	Indirect effect (physical activity), *β* (95% CI)	Indirect effect (depression), *β* (95% CI)	PM^c^
Learning (domain *z*-score)	0.93	–.68 (–.83, –.55)	–.13 (–.20, –.07)	–.04 (–.09, –.01)	–.09 (–.15, –.04)	19%
Memory (domain *z*-score)	0.92	–.70 (–.84, –.56)	–.12 (–.18, –.06)	–.04 (–.08, –.01)	–.08 (–.13, –.03)	17%
Verbal fluency (domain *z*-score)	0.91	–.23 (–.35, –.11)	–.07 (–.13, –.02)	–.04 (–.08, –.01)	–.03 (–.08, .01)	31%
Attention or working memory (domain *z*-score)	0.93	–.22 (–.31, –.13)	–.02 (–.06, .01)	.001 (–.02, .02)	–.02 (–.06, .006)	11%
Processing speed (domain *z*-score)	0.92	–.61 (–.84, –.41)	–.10 (–.22, –.02)	–.001 (–.05, .05)	–.10 (–.22, –.02)	17%
Executive function (domain *z*-score)	0.91	–.35 (–.53, –.17)	–.03 (–.13, .04)	.01 (–.03, .04)	–.04 (–.13, .03)	9%
Motor skills (domain *z*-score)	0.91	–.29 (–.55, –.13)	–.05 (–.13, .07)	–.01 (–.06, .04)	–.04 (–.10, .03)	18%
Global *z*-score	0.93	–.35 (–.44, –.26)	–.06 (–.10, –.03)	–.02 (–.04, .002)	–.04 (–.08, .02)	17%

^a^CFI: Comparative Fit Index.

^b^95% bias-corrected confidence intervals were presented (bootstrap sample size=2000), which did not include 0, showing the mediation effect was statistically significant (*P*<.05). The results were reported after controlling for significant background variables (*P*<.10) and other potential confounders.

^c^PM: percent mediated.

## Discussion

### Principal Results

Our results confirmed that older people living with HIV performed more poorly in global and all domains of neruocognitive performance compared to HIV-negative controls. The prevalence of neurocognitive impairment may be high among older people living with HIV in China [56]. Integrating prevention, screening, and management of neurocognitive impairment with existing HIV services is hence important for older people living with HIV in China.

Older people living with HIV had more severe depressive symptoms compared with their HIV-negative counterparts. Such finding was consistent with those from previous studies [57-59]. As compared to HIV-negative controls, a higher proportion of older people living with HIV were unmarried or living alone. Such between-group differences might contribute to higher depression among older people living with HIV. Previous studies suggested that older adults who lived alone were more likely to report feeling of depression compared with those who live with a spouse or other family member [60]. Since the implementation of the *treat-all* policy, the overall ART coverage in China has increased sharply [61]. The target to have 90% of people living with HIV on ART to achieve viral suppression has been already achieved in China [61]. The life expectancy of people living with HIV in China will largely increase. It is time to pay more attention to improve the mental health well-being of people living with HIV. The Joint United Nations Program on HIV and AIDS proposes adding a 4th “90” to the HIV testing and treatment target, which is to have 90% of people living with HIV with viral load suppression to have good health-related quality of life [62]. However, there is a dearth of mental health services targeting older people living with HIV in China. Improvements are greatly needed.

Consistent with previous studies among people infected with HIV [11,29,30] and HIV-negative populations [28], more severe depressive symptoms were negatively associated with neurocognitive function. Our findings suggested that, among older people aged ≥50, deficits in learning, memory, and processing speed were sensitive to depressive symptoms. Since depression is modifiable through interventions, mental health promotion will contribute to preventing or slowing down the progression of neurocognitive impairment among older individuals. Given that pharmacological treatment (antidepressant medication) may negatively affect neurocognitive function [63], psychological interventions may have a priority. Positive psychological interventions are recommended because they have some advantages compared to traditional psychological interventions, such as being less dependent on psychologists or psychiatrists and having longer effects [64,65]. They are potentially suitable in resource-limiting regions such as China.

Older people living with HIV have lower levels of physical activity compared with HIV-negative controls, as they may have more barriers to perform physical activities, probably due to HIV-positive status. Consistent with the findings of previous studies [33,66], higher levels of physical activity were associated with better neurocognitive performance among older individuals, especially in domains such as learning as well as memory and verbal fluency. Previous studies have shown that Tai chi resulted in greater improvements in neurocognitive function compared to the attention-control groups, and Western exercises including aerobics incorporated endurance, resistance or strength, and flexibility exercises [67]. Since Tai chi is slow and gentle, it is suitable for older individuals. It is also highly acceptable by the Chinese population. Health workers should consider promoting Tai chi to prevent or slow down neurocognitive impairment among both HIV-positive and HIV-negative older individuals.

Depressive symptoms and level of physical activity partially mediated the associations between HIV status and global and four domains of neurocognitive function. It suggested that change in mental health and physical activity after HIV infection may partially explain why older people living with HIV are more susceptible to neurocognitive impairment. Therefore, promoting mental health well-being and physical activity are potential entry points to slow down the progress of neurocognitive impairment among older people living with HIV and should be incorporated into routine care for this group. Future studies should explore factors associated with depressive symptoms and physical activities among older people living with HIV in China to develop culturally appropriate interventions.

### Limitations

The strengths of this study included the use of comprehensive neurocognitive tests and well-matched HIV-negative controls. However, it also had some limitations. First, the cross-sectional study design limited the ability to establish the causality of depressive symptoms or physical activities on neurocognitive functions. Second, we did not obtain sociodemographic characteristics of individuals who refused to participate in the study, and hence were not able to compare the difference in these characteristics between participants and nonparticipants. A selection bias thus might exist. Third, since the participants all came from 1 Chinese city, caution should be taken when generalizing the results to older people living with HIV in China. Fourth, we did not measure high-risk behaviors among the study participants. Fifth, we did not measure survey satisfaction in this study. Moreover, we did not measure anxiety, another important psychological well-being indicator, in this study. Furthermore, because some exclusion criteria and disease conditions were based on self-reported data, reporting bias might exist. Finally, this study only used 1 test to measure the information processing speed (Chinese Trail Making Test Part A) and executive function (Chinese Trail Making Test Part B).

### Conclusions

Older people living with HIV performed more poorly in global and all specific domains of neruocognitive performance compared with the HIV-negative controls. They also reported more severe depressive symptoms and lower levels of physical activity compared with their HIV-negative counterparts. Depressive symptoms and level of physical activity partially mediated the associations between HIV status and neurocognitive function. Change in mental health and physical activity after HIV infection may partially explain why older people living with HIV are more susceptible to neurocognitive impairment. Promoting mental health well-being and physical activity are potential entry points to slow down the progress of neurocognitive impairment among older people living with HIV.

## Appendix

Multimedia Appendix 1 Path analysis based on raw scores of neurocognitive test.

## Abbreviations

ART: antiretroviral therapy
CD4: cluster of differentiation 4
CES-D-20: 20-item Center for Epidemiological Studies-Depression
MET: metabolic-equivalent task
