# Supplementary material for: The Role of Depressive Symptoms and Physical Activity Levels in Mediating the Association Between HIV Status and Neurocognitive Functions Among Individuals Aged at Least 50 Years in China: Cross-sectional Study
Source: JMIR Public Health Surveill. 2022 Aug 19;8(8):e32968. doi: 10.2196/32968 (PMC9440416; doi:10.2196/32968)
Supplement: Multimedia Appendix 1 [file publichealth_v8i8e32968_app1.pdf]

Multimedia appendix 1 Path analysis based on raw scores of neurocognitive test

Table S1. Model fit and indirect effects of the proposed mediation model when using raw score

Dependent variable	CFI	Total effect β (95%CI)	Indirect effects β (95%CI)	Indirect effects (physical activity) β (95%CI)	Indirect effects (depression) β (95%CI)	PM
CAVLT–Total learning (raw score)	.92	–6.40 (–7.81, –5.12)	–1.21 (–1.85, –.69)	–.37 (–.82, –.08)	–.84 (–1.38, –.35)	19%
CAVLT–Delayed recall (raw score)	.92	–2.00 (–2.42, –1.58)	–.31 (–.50, –.15)	–.10 (–.22, –.01)	–.21 (–.38, –.06)	15%
Verbal fluency (raw score)	.92	–2.03 (–3.10, –.97)	–.63 (–1.14, –.18)	–.34 (–.68, –.10)	–.29 (–.68, .08)	31%
Digit span (raw score)	.91	–1.26 (–1.81, –.73)	–.20 (–.43, .02)	–.01 (–.15, .12)	–.19 (–.40, –.02)	16%
Visual span (raw score)	.93	–.66 (–1.06, –.24)	–.03 (–.20, .16)	.01 (–.10, .11)	–.03 (–.18, .13)	4%
CTMT–A [a] (raw score)	.94	6.93 (4.65, 9.38)	1.07 (.06, 2.28)	–.01 (–.58, .51)	1.08 (.12, 2.31)	16%
CTMT–B [a] (raw score)	.93	33.83 (17.59, 51.10)	2.53 (–4.04, 1.44)	–1.11 (–4.56, 2.20)	3.64 (–2.45, 11.75)	1%
Dominant hand (raw score)	.92	9.38 (3.39, 15.97)	4.01 (1.54, 7.00)	.88 (–.84, 2.89)	3.13 (1.03, 5.58)	43%
Nondominant hand (raw score)	.90	14.00 (5.47, 31.45)	.37 (–8.46, 4.91)	.19 (–3.48, 2.70)	.11 (–5.10, 3.19)	0%

CFI: Comparative Fit Index

PM: Percent mediated.

95% Bias–corrected confidence intervals (95% CI) were presented (bootstrap sample size = 2000), which did not include 0 showing the mediation effect was statistically significant (*P*<.05). Results were reported after controlling for significant background variables (*P*<.10) and other potential confounders.

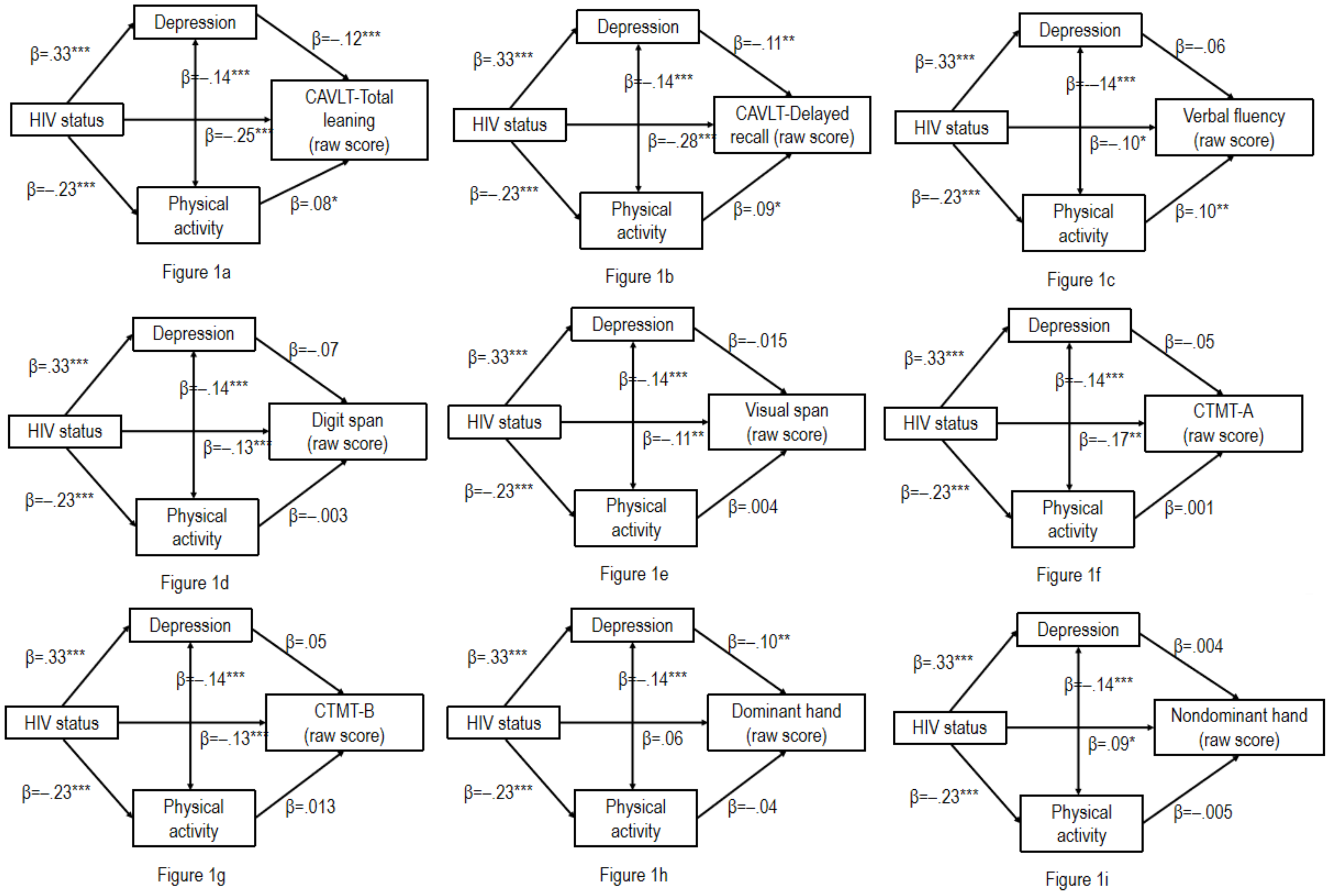

Figure S1 Mediation effects of physical activity and depressive symptoms in the association between HIV status and cognitive function (raw score)

*: $p < 0.05$; **: $p < 0.01$; ***: $p < 0.001$

The path analysis presented the standardized regression weights and P value of each path.

CAVLT-TL, Chinese Auditory Verbal Learning Test-total learning; CAVLT-DR, Chinese Auditory Verbal Learning Test-total learning-delayed recall; CTMT-A, Chinese Trail Making Test Part A; CTMT-B, Chinese Trail Making Test Part B
